# Multiple symmetric lipomatosis with secondary laryngeal obstruction: A case report

**DOI:** 10.1097/MD.0000000000021014

**Published:** 2020-07-02

**Authors:** Yu Cui, Xiangyan Cui, Shengrui Gao, Zhanpeng Zhu, Wanzhong Yin

**Affiliations:** a Departments of Otolaryngology; b Departments of Neurosurgery, The First Hospital of Jilin University, Changchun, Jilin, P.R. China.

**Keywords:** laryngeal obstruction, larynx, Madelung disease, multiple symmetric lipomatosis

## Abstract

**Introduction:**

Multiple symmetric lipomatosis (MSL) is an uncommon medical condition characterized by symmetric fat accumulation mainly in the neck and other upper body regions. The involvement of the larynx is rare according to the literature, and we present a case of MSL with larynx involvement treated with a surgical approach.

**Patient concerns:**

A 55-year-old male was admitted to our hospital due to progressively aggravated breathing difficulty, and tracheotomy was performed before transfer. When he tried to block the cannula, the breathing difficulty returned. The patient's neck had been thickening for the past 2 years.

**Diagnosis:**

Pathological examination confirmed the diagnosis of MSL.

**Interventions:**

The patient underwent lumpectomy and neck exploration.

**Outcomes:**

The lipoma was removed, the patient was free of any dyspnea symptoms and recovered well, and the tracheal cannula was removed at a local hospital.

**Conclusion:**

MSL can infiltrate the larynx and grow into the preepiglottic space and paraglottic spaces, resulting in breathing difficulties. Lipomas present in the spaces described above must be removed at the same time; otherwise, symptoms of dyspnea cannot be alleviated.

## 1 Introduction

Multiple symmetric lipomatosis (MSL) is characterized by the presence of multiple nonencapsulated fatty infiltrations in the neck, shoulders, arms, and upper back.^[1]^ It was first described by Sir Benjamin Brodie in 1846.^[2]^ Later, the first series of 33 cases was reported by Otto Madelung in 1888,^[3]^ and the disease was characterized by Pierre Emile Launois and Raoul Bensaude in 1898.^[4]^ Therefore, in the literature, this disease has also been known as Madelung disease, Launois-Bensaude disease, and benign symmetric lipomatosis. MSL predominantly affects males over females at middle age with heavy alcohol consumption, especially in the Mediterranean.^[5,6]^ Although hypotheses have been raised regarding the association of the disease with ethanol, the underlying etiology remains unclear.

Although the main effect of MSL is cosmetic deformity and restricted neck movement, compression of the trachea or esophagus or, more rarely, infiltration of the larynx can occur with associated dyspnea, dysphonia or dysphagia. MSL with laryngeal involvement has only been reported in 10 cases in the literature,^[7–15]^ among which only 4 received surgical treatment.^[9,13,15]^ Herein, we present another case of MSL with laryngeal involvement treated with a surgical approach and review the literature on this topic. This study was approved by the Jilin University Ethics Review Board, and the patient provided informed consent for publication of the case.

## 2 Case report

A 55-year-old male was admitted to our hospital due to progressively aggravated breathing difficulty. Tracheotomy was performed at a local hospital before the patient was transferred, and the breathing difficulty was relieved, but the breathing difficulty returned immediately after trying to block the cannula. He had a 30-year history of alcohol abuse (>100 g ethanol per day). His medical history was otherwise unremarkable. His neck had been thickening for the past 2 years.

**Figure 1:**
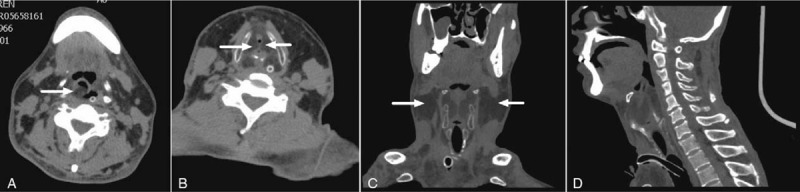
Preoperative computer tomography scan of the neck. A. Axial view showed marked infiltration of the right aryepiglottic fold by tissue with low density (white arrow). B. Axial view showed marked infiltration of bilateral paraglottic spaces by tissue with low density (white arrows). C. Coronal view showed bilateral subcutaneous infiltration in the neck (white arrows). D. Sagittal view show lipomas compression resulting in severe airway stenosis.

**Figure 2:**
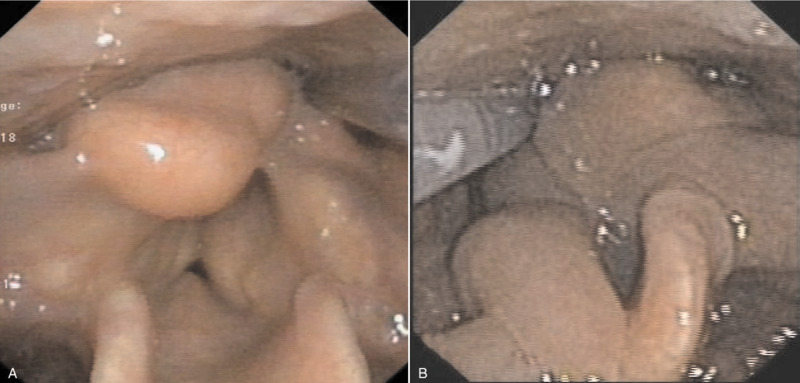
A, Preoperative laryngoscopy showed bilateral swollen ventricular bands in the supraglottis, obstructing the glottis. A smooth mucosal intact bulge was seen in the region of postcricoid and right arytenoid cartilage. B, Postoperative laryngoscopy (2 weeks after the surgery) showed mucosal swelling in the supraglottis, leaving the glottis invisible.

Physical examination revealed that the tracheal cannula was in place without obstruction. It was observed that there was symmetrical massive enlargement of the neck ventrally and dorsally on both sides. There were no palpable masses or tenderness in the affected region. The laboratory results indicated an elevated gamma-glutamyl transpeptidase level of 827 U/L and were otherwise unremarkable. Computed tomography of the neck (See Fig. 1) indicated a shallower right pyriform sinus, a decreased caliber of the lumen in the rima glottidis region, and massive infiltration in the right aryepiglottic fold, bilateral paraglottic spaces, and the neck by tissue with an attenuation value of -48 Hounsfield units. Laryngoscopic evaluation (See Fig. 2) revealed bilateral swollen ventricular bands (false vocal cords) in the supraglottis, which obstructed the glottis. A smooth mucosal intact bulge was seen in the postcricoid and right arytenoid cartilage regions. These factors all led to a diagnosis of MSL.

**Figure 3:**
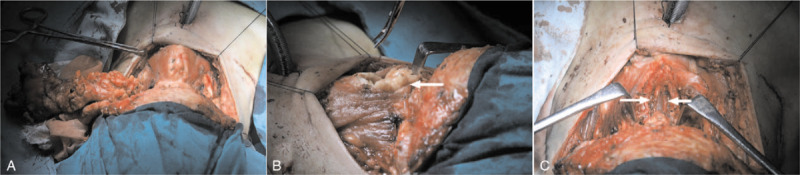
A, Lipotectomy was performed bilaterally in the neck. B, Pulling the infrahyoid muscles to the sides exposed marked lipomatous deposition on the surface of the thyroid cartilage and the preepiglottic space. C, Exploration revealed lipomatous deposition in the bilateral paraglottic spaces, and therefore lipotectomy was performed.

Once the diagnosis was made, the plan was for the patient to undergo lumpectomy and neck exploration. During the surgery, the superficial fatty masses of the neck were excised (See Fig. 3A) before exploration of the deeper anatomy was performed. Pulling the stripped muscles to the side exposed massive lipomatous lesions deposited on the surface of the thyroid cartilage and the preepiglottic space (See Fig. 3B). After resection was completed in the aforementioned region, it was noticed that the lipomatous lesions had grown into the anterior epiglottic space. Further exploration revealed more lesions in the bilateral paraglottic spaces, and accordingly, lipectomy was performed (See Fig. 3C). The postoperative pathological results were consistent with the diagnosis of MSL.

Two weeks after the surgery, laryngoscopy showed mucosal swelling in the supraglottis, leaving the glottis region invisible. Further follow-up was conducted 3 months after the surgery by telephone. The patient was free of any dyspnea symptoms and recovered well. The tracheal cannula was removed at a local hospital.

## 3 Discussion

MSL was characterized by Launois and Bensaude as diffuse multiple lipomatosis predominantly located symmetrically in the cervical region.^[4]^ Later, published case reports revealed that the disease may involve the neck, face, shoulder, upper extremities, upper dorsal regions, and even the tongue.^[16,17]^ Enzi introduced a classification for MSL in 1984.^[5]^ Type I, or Madelung collar, manifests as symmetrical fat accumulation around the nape of the neck, upper back, shoulders, and upper arms predominantly in males. These patients present as pseudoathletic with massive enlargement protruding from the skin. Type II, on the other hand, affects males and females equally, with diffuse fat distribution in the upper back, deltoid, upper arms, hips, and upper thigh region. These patients present with generalized obesity. Donhauser et al further divided type I disease into horse collar lipoma and pseudoathletic appearance.^[18]^

The pathogenesis of MSL remains obscure, even though a few theories have been hypothesized. Lipid metabolism malfunction has been noticed in these patients. Enzi reported that a significantly increased lipoprotein lipase activity in adipose tissue, plasma hyperalphalipoproteinemia and a particular defect of adrenergic-stimulated lipolysis in lipomatous tissue were found in these patients.^[5]^ More recently, a mitochondrial DNA damage theory was proposed by Berkovic et al.^[19]^ Emerging evidence suggests that mitochondrial dysfunction may depress the lipolytic pathway.^[20,21]^ Approximately 60% to 90% of MSL patients have chronic alcoholism.^[22]^ Alcohol consumption may contribute to adipocyte hyperplasia considering its effects on lipogenesis, antilipolysis, lipid oxidation reduction, and mitochondrial metabolism.^[23]^

The diagnosis of MSL is fairly easily made and is mainly established by physical examination due to the typical appearance of these patients. In most cases, cosmetic deformity can be the only complaint from patients. However, patients that present with dyspnea, dysphonia, or dysphagia have been reported. These symptoms are caused by direct compression in most scenarios^[1,23]^ and rarely by infiltration of the larynx.^[7–15]^ Imaging studies including computed tomography and magnetic resonance imaging can be helpful in these cases of airway obstruction to eliminate other possibilities^[24,25]^ and to identify the region of infiltration by the fatty tissue for later surgical reference. Fine-needle biopsy is usually not recommended, but there is still concern about imaging studies failing to differentiate the disease from malignant disease.^[26]^

To date, surgical resection and suction-assisted lipectomy are the only effective and optimal treatments for MSL. Considering the infiltrative nature of lipomas and the high recurrence rate of the disease,^[1,27]^ the aim of the surgery is to reduce but not completely remove the lipomas. However, abstinence from alcohol may prevent progression in the size of the fatty tissues.^[28]^ When lipomas infiltrate the larynx and grow into the preepiglottic space and paraglottic spaces, which results in dyspnea, lipomas in the spaces described above must be removed at the same time; otherwise, symptoms of dyspnea cannot be alleviated. Salbutamol might be useful, but the efficacy of medical treatment is still unclear due to inconsistent results.^[27]^ Abstinence from alcohol is recommended in these patients. Even though the impact may be minimal, it has been reported that alcohol abstinence might reduce the recurrence rate of the disease.^[1]^ We present a case of MSL with laryngeal involvement treated by surgical lipectomy, which is rare according to the literature. This patient developed dyspnea at an early stage and required tracheotomy, which was an indication for surgical intervention. This might be the most severe case of MSL reported so far.

In conclusion, the main effect of MSL is cosmetic deformity and restricted neck movement. The aim of surgery is to reduce but not completely remove the lipomas. However, when lipomas infiltrate the larynx and grow into the preepiglottic space and paraglottic spaces, which results in dyspnea, lipomas in the spaces described above must be removed at the same time; otherwise, symptoms of dyspnea cannot be alleviated.

## Author contributions

**Resources:** Xiangyan Cui.

**Supervision:** Shengrui Gao.

**Writing – original draft:** Yu Cui.

**Writing – review & editing:** Zhanpeng Zhu, yin wanzhong.
